# Water on hydrophobic surfaces: mechanistic modeling of polyethylene glycol-induced protein precipitation

**DOI:** 10.1007/s00449-018-2054-5

**Published:** 2018-12-07

**Authors:** Steffen Großhans, Gang Wang, Jürgen Hubbuch

**Affiliations:** Karlsruhe Institute of Technology (KIT), Institute of Process Engineering in Life Sciences Section IV: Biomolecular Separation Engineering, 76131 Karlsruhe, Germany

**Keywords:** Preparative protein precipitation, Polyethylene glycol, Mechanistic modeling, Water structure, Industrial monoclonal antibody

## Abstract

For the purification of biopharmaceutical proteins, liquid chromatography is still the gold standard. Especially with increasing product titers, drawbacks like slow volumetric throughput and high resin costs lead to an intensifying need for alternative technologies. Selective preparative protein precipitation is one promising alternative technique. Although the capability has been proven, there has been no precipitation process realized for large-scale monoclonal antibody (mAb) production yet. One reason might be that the mechanism behind protein phase behavior is not completely understood and the precipitation process development is still empirical. Mechanistic modeling can be a means for faster, material-saving process development and a better process understanding at the same time. In preparative chromatography, mechanistic modeling was successfully shown for a variety of applications. Lately, a new isotherm for hydrophobic interaction chromatography (HIC) under consideration of water molecules as participants was proposed, enabling an accurate description of HIC. In this work, based on similarities between protein precipitation and HIC, a new precipitation model was derived. In the proposed model, the formation of protein–protein interfaces is thought to be driven by hydrophobic effects, involving a reorganization of the well-ordered water structure on the hydrophobic surfaces of the protein–protein complex. To demonstrate model capability, high-throughput precipitation experiments with pure or prior to the experiments purified proteins lysozyme, myoglobin, bovine serum albumin, and one mAb were conducted at various pH values. Polyethylene glycol (PEG) 6000 was used as precipitant. The precipitant concentration as well as the initial protein concentration was varied systematically. For all investigated proteins, the initial protein concentrations were varied between 1.5 mg/mL and 12 mg/mL. The calibrated models were successfully validated with experimental data. This mechanistic description of protein precipitation process offers mathematical explanation of the precipitation behavior of proteins at PEG concentration, protein concentration, protein size, and pH.

## Introduction

Biologics represent a growing share of the pharmaceutical market, reaching global sales of USD 228 billion in 2016 [1]. Among them, monoclonal antibodies (mAb) are the most important family of products [2]. The fact that many mAbs have a relatively low potency requiring high doses makes mAbs among the most expensive drugs [3]. In 2015, the patent of first-generation mAbs began to expire, resulting in a number of biosimilars approved in the USA and Europe [4]. Together with an increasing pressure on health-care budgets, cost savings are desired [5]. Therefore, improvements in downstream processes such as alternative methods or novel development strategies are necessary [6].

Selective protein precipitation has been known as a cost-efficient alternative purification step for a long time [7]. Phase separation is carried out by adding precipitation agents, such as inorganic salts, organic solvents, or nonionic polymers to the protein solution [8–11]. Especially, the use of polyethylene glycol (PEG) is favorable as it is not reported to harm the protein by, for example, causing denaturation [12]. For some biopharmaceutical products, precipitation is already well established. For example, ethanol or PEG precipitation is the basis of the extraction of immunoglobulin G from human plasma [13]. Viral vaccines and virus-like particles (VLP) are purified or concentrated through PEG or salt precipitation [14, 15]. Although there are a lot of studies on precipitation of recombinant mAbs as well, it has not been implemented for large-scale mAb production yet [16–18].

For downstream process development (DSP), mechanistic understanding is needed to meet the demands of the quality by design (QbD) approach suggested by the US Food and Drug Administration (FDA) [19]. For precipitation, the understanding of protein phase behavior is mandatory [20]. Although protein phase behavior has been well investigated experimentally, the mechanism behind precipitation has not been completely understood. This leads to many degrees of freedom in process development and makes it challenging.

Modeling can reduce the number of experiments and lead to a more thorough process understanding [21]. Cohn *et al.* derived an equation to describe protein precipitation [22]. This equation is a useful empirical expression, but not much of a mechanistic description [23]. Its parameters were specified for salt precipitation and its application to precipitation using polymers was shown [24, 25]. Sim *et al.* generalized the model on the basis of hydrodynamic radii [26]. Quantitative structure–activity relationship (QSAR) modeling was used to estimate the parameters of the Cohn equation [27]. Anyhow, all these models have difficulty dealing with small molecular weight proteins and are not capable to cover variations in the initial protein concentration.

PEG-induced precipitation can be described by the theory of excluded volume [28–30]. According to this theory, adding a certain amount of PEG to a protein solution results in a phase transition of the proteins. The polymers are reported to trap the solvent and, therefore, sterically exclude proteins from solvent regions occupied by the polymers. In other words, the polymers and proteins compete for the solvent which they are solved in. The theory of attractive depletion introduces osmotic pressure as additional force in the process of PEG-induced protein precipitation [31, 32]. According to this theory, the polymer is sterically excluded from the surface of the protein, the depletion zone. The overlapping of two depletion zones causes a concentration gradient which leads to the described osmotic pressure. In both theories, there is no interaction between PEG and the proteins described; hence, all other forces between particles are still valid. Electrostatic interaction is known to have mainly repulsive influence on protein–protein interaction, so that attractive forces can be reduced to hydrophobic effects [33]. On the molecular level, water molecules next to hydrophobic surfaces are thought to have a well-ordered structure, as opposed to the bulk-like ordered water in free solution [34–36]. During precipitation, a rearrangement of the protein occurs and the solvent-accessible hydrophobic surface area is reduced. Simultaneously, the water structure has to be reorganized. A new equilibrium between well-ordered and bulk-like water is reached, resulting in an increased entropy [37, 38]. The hydrophobic effect described above is thought to occur between two proteins in precipitation, as well as between protein and ligand in hydrophobic interaction chromatography (HIC). This means that the mechanism which causes a protein to be retained on an HIC column is analogous to the mechanism that causes proteins to stick together and precipitate. Similarities between HIC and precipitation have been already shown. Melander and Horváth investigated the salt influence on the hydrophobic effect in precipitation and HIC [39]; Nfor and coworkers studied the interrelation between the number of released water molecules in protein precipitation and HIC [40]; Baumgartner and coworkers investigated the retention behavior during HIC and its correlation to protein–protein interaction [41]. The mechanistic effects of HIC are well investigated [42–44].

**Fig. 1:**
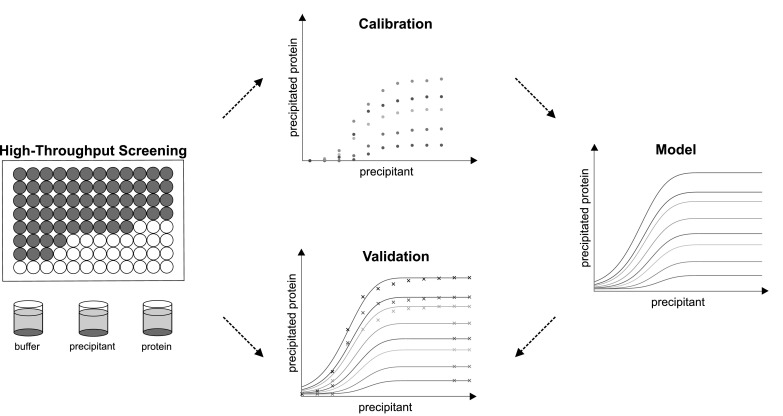
Mechanistic protein precipitation modeling. By varying the amount of buffer, protein and precipitant stock solutions, the precipitant and protein concentration were varied in high-throughput experiments. After phase separation, the protein concentration was detected using UV 280 measurement. 50 Data points were used as calibration set. With this data, the parameters of the model were estimated. The so-generated model was validated with the other 46 data points of the experimental data

In this study, a mechanistic protein precipitation isotherm model was derived, inspired by recent results in HIC modeling [45]. Based on existing precipitation theories, water was introduced as an additional component for the model building. With the help of high-throughput precipitation experiments, data for lysozyme, myoglobin, bovine serum albumin (BSA), and a mAb were generated. For each protein, 50 data points were used to calibrate the model. The three highest protein and the two highest PEG concentrations were excluded and afterward used as validation set (Fig. 1). The model predictability for a wide range of properties such as size, hydrophobicity, and the isoelectric point (pI) was shown.

## Theory

In 1925, Cohn and coworkers introduced an afterward widely accepted semi-logarithmic equation for modeling protein precipitation:

1 $$\begin{aligned} \mathrm{log}(S) = S_0-m \alpha . \end{aligned}$$

The useful empirical equation describes the protein solubility *S* in mg/mL in the presence of *m* PEG in % (w/w). Here, the phase behavior of a constant protein concentration depends on the precipitation efficiency $\alpha$ and the protein solubility in the absence of PEG $S_0$. This equation represents a summary of a macroscopic observation of the precipitation behavior.

In the present work, the focus is placed on a mechanistic level, especially on the behavior of water molecules during protein precipitation. In the model assumption, the precipitation mechanism of *n* protein molecules *P* by a PEG molecule PEG forming the precipitate $P_{p}$ is considered. The hydrophobic surfaces of proteins are thought to be stabilized by well-ordered water molecules. The precipitate $P_{p}$ is assumed to be stabilized by $\beta$ bulk-like ordered water molecules $W_{B}$:

2 $$\begin{aligned} nP + \mathrm{PEG} \rightleftharpoons P_{p} + n\beta W_{B}. \end{aligned}$$

Precipitation using PEG is known to be a fast process [46]. Investigations by Atha and Ingham showed that longer incubation times did not have an influence on the precipitation behavior [25]. Thus, the following equilibrium was considered:

3 $$\begin{aligned} \varDelta \mu = \mu _{P_{p}} + n\beta \mu _{W_{B}} - n\mu _{P} - \mu _{\mathrm{PEG}}. \end{aligned}$$

The chemical potentials $\mu$ are deduced to apply this constraint. It was assumed that the protein surface charges can be considered negligible in the present hydrophobically driven mechanism.

Considering the equilibrium $\varDelta \mu = 0$ at constant temperature and pressure, it is

4 $$\begin{aligned}&RT\ln x_{P_{p}}\gamma _{P_{p}} + n\beta RT\ln x_{W_{B}}\gamma _{W_{B}} - nRT\ln x_{P}\gamma _{P}\nonumber \\&\qquad - RT\ln x_{\mathrm{PEG}}\gamma _{\mathrm{PEG}} \end{aligned}$$

5 $$\begin{aligned}&\quad = -\mu _{P_{p}}^{0} - n\beta \mu _{W_{B}} + n\mu _{P} + \mu _{\mathrm{PEG}} \end{aligned}$$

6 $$\begin{aligned}&\quad = -\varDelta G^{0} = RT \ln K. \end{aligned}$$

To simplify the model equation, the activity coefficients of protein, PEG, and precipitate are assumed to be a constant. The equilibrium constant *K* is derived as

7 $$\begin{aligned} K = \frac{x_{P_{p}}x_{W_{B}}^{n\beta }}{x_{P}^{n}x_{\mathrm{PEG}}} \Rightarrow K = \frac{q a_{W_{B}}^{n\beta }}{c_{P}^{n}c_{\mathrm{PEG}}}, \end{aligned}$$

with *q* and $c_{P}$ being the precipitated protein and protein in solution, respectively. $c_{\mathrm{PEG}}$ depicts the concentration of PEG in solution, and $a_{W_{B}}$ the activity of the bulk-like ordered water molecules. In the following step, parameterizations for $a_{W_{B}}$ and $\beta$ have to be found. According to stoichiometric considerations, the number of water molecules involved is linearly correlated to the precipitated protein *q*. Thus, a linear correlation is proposed to substitute $a_{W_{B}}$:

8 $$\begin{aligned} a_{W_{B}}^{n\beta }\cong & {} \nu q^{n\beta }. \end{aligned}$$

The stoichiometric constant $\nu$ is assumed to be independent of the PEG concentration. Inserting Eq. 8 into Eq. 7, and collecting all constants on the left-hand side, the following isotherm equation is obtained:

9 $$\begin{aligned} K= & {} \frac{\nu q^{1+n\beta }}{c_{p}^{n}c_{\mathrm{PEG}}} \end{aligned}$$

10 $$\begin{aligned} {\mathop {\Longleftrightarrow :}\limits ^{\cdot \frac{1}{\nu }}} k_{eq}= & {} \frac{q^{1+n\beta }}{c_{p}^{n}c_{\mathrm{PEG}}}. \end{aligned}$$

Finally, the PEG and protein dependency of the bulk-like ordered water molecules $\beta$ is modeled. Since hydration of PEG, protein, and salt ions has high similarities, e.g., attracting water molecules to form a hydration shell, the model originally describing the hydration number of the salt ions *h* is employed [47]:

11 $$\begin{aligned} h = h_{0}\exp (-kc_{s}), \end{aligned}$$

where $h_{0}$ is the ionic hydration number at infinite dilution and *k* the constant that accounts for the dependency of the hydration number on the ionic concentration $c_{s}$. $\beta$ and *h* are assumed to be reciprocal, so that the model parameter $\beta$ can be approximated by the exponential term:

12 $$\begin{aligned} \beta = \beta _{0} \exp (\beta _{1}c_{\mathrm{PEG}}+\beta _{2}c_{p_0}), \end{aligned}$$

where $\beta _{0}$ is the hydration number at infinite dilution of PEG and protein, whereas $\beta _{1}$ and $\beta _{2}$ are the constants that account for the dependency of the hydration number on PEG and initial protein concentration, respectively. This completes the derivation of the equilibrium formulation of the precipitation isotherm model.

## Materials and methods

### Disposables

All precipitation experiments were carried out in 350 $\mu L$ polypropylene flat bottom 96-well micro plates (Greiner Bio-One, Kremsmünster, Austria). For spectroscopic measurements, samples were diluted into Greiner UV-Star$^\circledR$ micro plates (Greiner Bio-One, Kremsmünster, Austria).

### Chemicals and stock solutions

As buffer substances, sodium hydrogen carbonate and tris(hydroxymethyl)-aminomethane (both Merck KGaA, Darmstadt, Germany) were used. Tris–hydrochloride was obtained from PanReac AppliChem (Darmstadt, Germany). Sodium carbonate was obtained from Sigma-Aldrich (St. Louis, MO, USA). The PEG with a median molecular mass of 6000 was obtained from Merck KGaA (Darmstadt, Germany). All buffers were prepared with a concentration of 50 *mM*. For this, the appropriate amounts of associated buffer components were weighed and dissolved in $ddH_{2}O$. The desired pH was achieved by varying the amount of acid and basic component for each buffer. For the 40 $\%$ (w/w) PEG 6000 and 50 $\%$ (w/w) PEG 6000 stock solution, the buffer components were first dissolved in $ddH_{2}O$ followed by adding the appropriate amount of PEG 6000.

### Preparation of protein stock solutions

Lysozyme from chicken egg white was purchased from Hampton Research (Aliso Viejo, CA, USA). Myoglobin and BSA were purchased from Sigma-Aldrich (St. Louis, MO, USA). The mAb was provided as purified mAb from LEK d.d. (Ljubljana, Slovenia). Lysoszyme, myoglobin, and BSA were provided as lyophilized powder and, therefore, first dissolved in the appropriate buffer. Afterward, all proteins including the mAb were filtered using 0.2 $\mu m$ cellulose acetate syringe filters (Satorius, Göttingen, Germany). Following the filtration, proteins were rebuffered and desalted into the associated buffer using PD 10 desalting columns (GE Healthcare, Little Chalfont, UK).

### Generation of precipitation curves

All precipitation experiments were carried out on a Tecan Freedom Evo 200 System liquid handling station (Tecan, Männedorf, Switzerland). The liquid handling station was equipped with an 8-tip liquid handling arm, a robotic manipulator arm, a Te-Shake orbital shaker, an Infinite$^\circledR$ 200 UV–Vis spectrophotometer (all Tecan, Männedorf, Switzerland), and a Rotanta 46RSC centrifuge (Hettlich GmbH & Co. KG, Tuttlingen, Germany). The system was controlled by Evoware 2.5 (Tecan, Männedorf, Switzerland). Excel 2016 (Microsoft, Redmond, WA, USA) was used as data import format and for data storage. All calculations were done using Matlab$^\circledR$ R2016a (The Mathworks, Natick, MA, USA). All experiments were carried out at 20$^\circ C$, controlled by air-conditioning. Systems with a total volume of 250 $\mu L$ containing varying protein and PEG concentrations were prepared. The PEG concentration was varied in 12 equidistant steps. The protein concentration was varied from 1.5 *mg* / *mL* to 12.0 *mg* / *mL* in 12 steps. The position for each system on the 96-well micro plate was randomized. After adding the protein stock solution, the system was incubated for 15 *min* on the orbital shaker at 1000 *rpm*, followed by 15 *min* without shaking. To analyze the amount of precipitated protein, the microplate was centrifuged for 30 *min* at 3400*g*. Then, the supernatant was sampled and diluted at a ratio of 1:6 for lysozyme, 1:3 for BSA, and 1:4 for myoglobin and the mAb. Subsequently, UV–Vis absorption at 280 *nm* was measured. The protein concentration was calculated based on a linear calibration curve. For data pretreatment, the percentage standard error for each triplicate was calculated. In case of deviations larger than 5 %, possible outliers were eliminated.

### Numerical procedures

The equilibrium precipitation isotherm model Eqs. 10 and 12 proposed in the previous section contain unknown parameters, which cannot be determined directly. Model calibration and simulation were carried out in Matlab$^\circledR$ R2018a (The Mathworks, Natick, MA, USA). To solve the nonlinear equation, *fsolve* was used. The heuristic algorithm-simulated annealing *simulannealbnd* was employed to deliver the parameter estimates for $k_{eq}$, *n*, $\beta _{0}$, $\beta _{1}$, and $\beta _{2}$.

The data generated in high-throughput experimentation were split into calibration data and validation data. For model calibration, the three highest protein and the two highest PEG concentrations were excluded. The remaining 50 data points were used to prove the descriptive capability and accuracy of the suggested model. The other 46 data points, used as validation set, were compared with the model prediction to back up the model’s accuracy. This data were out of calibration range and therefore also showed the potential of the model to expand the predictability of the model.

## Results and discussion

### Model calibration

All precipitation data from high-throughput experimentation were divided into two data sets. The calibration data sets contained the five lowest protein and the ten lowest PEG concentrations. With the selection of the calibration data sets, the model predictability out of the calibration range should be proved. For paramter estimation, simulated annealing with the calibration data sets was used. The equilibrium coefficient $k_{eq}$, the number of proteins affected by one PEG molecule *n*, the hydration number at infinite dilution $\beta _0$, and the constants accounting for the dependency of hydration number on PEG concentration $\beta _1$ and protein concentration $\beta _2$ for lysozyme, myoglobin, BSA, and mAb at pH 7.5 and pH 8.5 are given in Table 1. Here, the natural logarithm of the $k_{eq}$ is presented for a better overview.

**Table 1:** Parameters of the precipitation model estimated from the calibration high-throughput experimental data. The natural logarithm of the equilibrium coefficient $k_{eq}$ is presented for a better overview

Parameter	Lysozyme	Myoglobin	BSA	mAb 7.5	mAb 8.5
$\ln {k_{eq}}$ $[-]$	34.71	15.41	9.78	22.53	19.83
*n* $[-]$	5.55	3.06	2.60	3.65	3.22
$\beta _{0}$ $[-]$	6.92 $\,\times \,10^{-3}$	4.30 $\,\times\, 10^{-4}$	2.74 $\times 10^{-3}$	1.46 $\times 10^{-2}$	3.77 $\,\times\, 10^{-2}$
$\beta _{1}$ [L/mol]	9.29 $\times 10^{1}$	1.18 $\times 10^{2}$	1.40 $\times 10^{2}$	2.37 $\,\times\, 10^{2}$	2.50 $\,\times\, 10^{2}$
$\beta _{2}$ [L/mol]	2.06 $\,\times\, 10^{3}$	1.51 $\,\times\, 10^{3}$	1.58 $\,\times\, 10^{3}$	1.05 $\,\times\, 10^{4}$	6.61 $\,\times\, 10^{3}$

**Fig. 2:**
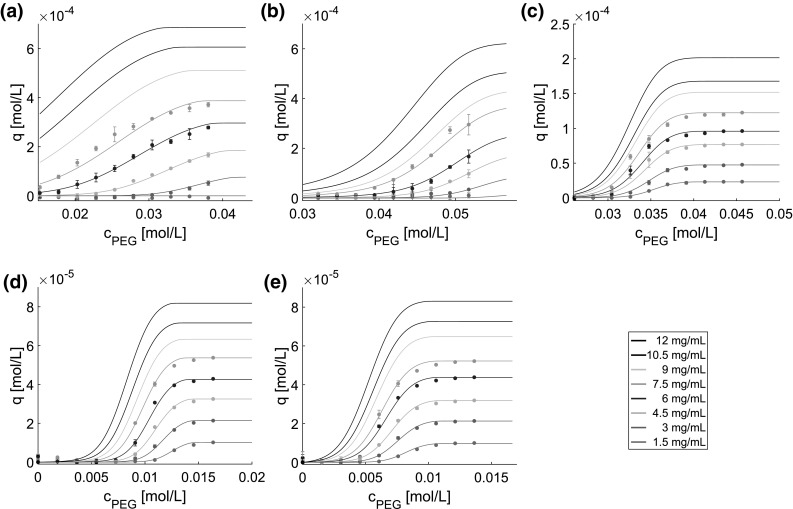
Comparison of model prediction (solid lines) and high-throughput experimental data used for model calibration (dots). Data points represent mean values of at least triplicates. **a** represents lysozyme, **b** myoglobin, **c** BSA, **d** mAb at pH 7.5, and **e** mAb at pH 8.5

Figure 2 shows the model simulation as solid curves and the experimental data used for model calibration as dots for lysozyme (Fig. 2a), myoglobin (Fig. 2b), BSA (Fig. 2c), mAb at pH 7.5 (Fig. 2d), and pH 8.5 (Fig. 2e). In all cases, the results cover eight protein concentrations between 1.5 *mg* / *mL* and 12 *mg* / *mL* with equidistant steps of 1.5 *mg* / *mL*. The investigated range of PEG concentrations varies according to the precipitation behavior of each protein. For lysozyme, myoglobin, and BSA, PEG concentrations of up to 0.056 mol/L are shown. The mAb precipitated at lower PEG concentration, so that PEG concentrations of up to 0.02 mol/L are presented.

**Fig. 3:**
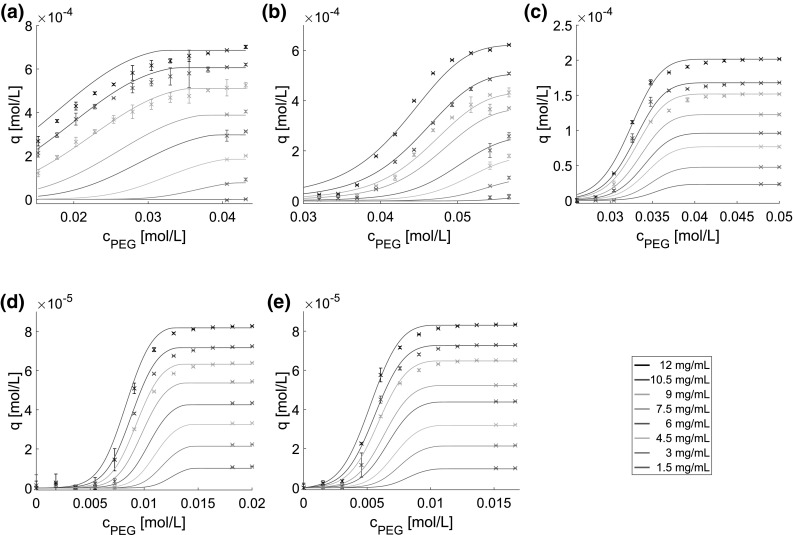
Comparison of model prediction (solid lines) and high-throughput experimental data used for model validation (crosses). Data points represent the mean values of at least triplicates. **a** represents lysozyme, **b** myoglobin, **c** BSA, **d** mAb at pH 7.5, and **e** mAb at pH 8.5. The solid lines are identical with Fig. 2 and are shown here for comparability

Despite the very different protein characteristics such as size, hydrophobicity, and surface charge distribution, the calibration data sets are described by the precipitation model accurately in all cases. Regardless of the differences in experimental conditions such as PEG concentration and pH value, a good identifiability of precipitation model parameters is observed. As the smallest protein investigated, lysozyme (14.6 *kDa*) shows the highest number of proteins affected by one PEG molecule with $n=$ 5.55. The slightly larger protein myoglobin (17.0 *kDa*) shows lower *n* with 3.06. For all other proteins, the *n* values were similar to those of myoglobin.

The hydration number constants $\beta _0$, $\beta _1$, and $\beta _2$ of each protein influence the $\beta$ function in the same order of magnitude. $\beta _1$ accounts for the hydration of PEG. Consequently, the behavior can be reconciled with the theory of excluded volume. By adding PEG, the accessible water for the protein is reduced. As this exclusion is caused by a steric phenomenon, the influence can be attributed to protein size, when the PEG species is kept the same. A linear correlation between $\beta _1$ and the molecular weight is observed in accordance with the linear correlation of precipitation behavior and the hydrodynamic radius of the protein for PEG as precipitant reported by Sim *et al.* [26]. The similarity of the $\beta _1$ values determined for the mAb at pH 7.5 and pH 8.5 supports this assumption. Furthermore, Hämmerling *et al.* confirmed this assumption, but pointed out the influence of additional factors, such as protein shape and other surface characteristics [27].

In the case of mAb, shifting the experimental conditions closer to their pI (pH 8.3–8.5) to pH 8.5 from pH 7.5 leads to decrease of *n*, and $k_{eq}$ and an increase of $\beta _{0}$, resulting in precipitation of mAb at lower PEG concentrations. At the same time, an increase in $\beta _2$ was observed. While $\beta _1$ could be assigned to the protein size, the nature of $\beta _0$ and $\beta _2$ appears to be more complex. The pH dependence of these parameters suggests a correlation to the surface characteristics of protein. The pH dependency of the hydration number $\beta _0$ is consistent with the results delivered by Xia and coworkers [48] suggesting that the water release increases as the buffer pH approaches the protein’s pI in HIC. The hydration number parameter $\beta _2$ is attributed to the influence of the protein concentration. Closer to the pI, this influence appears to be less important. In the absence of electrostatic interactions, the well-ordered water conformation is reported to be less stable and, therefore, it is favored to set free well-ordered water molecules [49].

### Model validation

To assess the predictive power of the calibrated precipitation models, the validation data sets were used. Precipitation processes using PEG as precipitation agent come with the drawback of highly viscous PEG solution, especially if PEG has to be added as liquid stock solution. In contrast to laboratory-scale process development or production, the addition of solid PEG is no option for high-throughput screenings. Furthermore, the availability of highly concentrated protein stock solutions is not always given. Hence, using a calibration set containing low protein and PEG concentration and using the model for expanding the response in both directions can simplify process development and give new options to the production process.

Figure 3 shows the model prediction as solid curves and the experimental data excluded from model calibration as crosses for lysozyme (Fig. 3a), myoglobin (Fig. 3b), BSA (Fig. 3c), and mAb at pH 7.5 (Fig. 3d) and pH 8.5 (Fig. 3e). A very good prediction can be found especially for myoglobin, BSA, and mAb at both pH values, and for lysozyme at low protein and, respectively, or at high PEG concentration. For lower PEG concentrations, however, the models tend to overestimate the amount of precipitated protein for all tested proteins. For 12 *mg* / *mL* lysozyme, the model overestimated the precipitation behavior over the examined PEG range. The root mean square errors of prediction (RMSEP) were 3.22 $\,\times\,10^{-5}\,\mathrm{mol/L}$ for lysozyme, 3.02 $\,\times\,10^{-5}$ mol/L for myoglobin, 7.51 $\,\times\,10^{-6}$ mol/L for BSA, 3.62 $\,\times\,10^{-6}$ mol/L for mAb at pH 7.5, and 2.71 $\,\times\,10^{-6}$ mol/L for mAb at pH 8.5.

Although simplifications and assumptions were made during the precipitation model derivation to balance accuracy with ease of use, predictability and validity are backed up by high-throughput experimental data of very different proteins under diverse operating conditions. The main difference between the widely used models, such as Cohn equation and models related to it, and the proposed precipitation model is its mechanistic nature and capability to describe the protein precipitation process dependent on both PEG and protein concentration.

The validation data showed the predictability of the model even out of the calibration range. This allows a easier and faster model calibration. Furthermore, it opens the possibility of using the model for prediction and control of processes with variations in the protein feed concentration.

## Conclusion

In the presented work, a mechanistic model for protein precipitation behavior with PEG was introduced by considering the insights into the water structure on a hydrophobic surface. The present approach proposes the equilibrium between well-ordered and bulk-like ordered water molecules on the hydrophobic surfaces of protein as the driving force for the precipitation process. This equilibrium is described for a constant buffer composition, in particular without change in pH and PEG type.

The model predictability could be proven for the proteins lysozyme, myoglobin, BSA, and mAb at pH 7.5, and the same mAb at pH 8.5 using validation data. The validation data were not included in the model building and beyond the calibration space. The model predictability could be found to be reasonable for proteins investigated despite their differences in properties such as size, hydrophobicity or charge of the proteins. The estimated model parameters lead to insights into the precipitation process itself, i.e., the mathematical explanation of proteins’ precipitation behavior dependent on their size, pH, and hydration.

In further studies, the dependency of model parameters on changes in pH and PEG type should be investigated to enhance the mechanistic understanding of protein precipitation with PEG. The applicability of the suggested model to protein precipitation with salt-induced precipitation could be tested in a systematic manner. Multi-component systems such as harvested cell culture fluid should be described to enable model-based optimization of selective precipitation processes.
